# Are nanophotonic intermediate mirrors really effective in enhancing the efficiency of perovskite tandem solar cells?

**DOI:** 10.1515/nanoph-2024-0658

**Published:** 2025-04-09

**Authors:** Kwangjin Kim, Jieun Lee, Jaewon Lee, Jin-Young Kim, Hae-Seok Lee, Seungwoo Lee

**Affiliations:** KU-KIST Graduate School of Converging Science and Technology, 34973 Korea University, Seoul, 02841, Republic of Korea; Department of Materials Science and Engineering, Seoul National University, Seoul, 08826, Republic of Korea; Graduate School of Energy and Environments (Green School), Korea University, Seoul, 02841, Republic of Korea; Department of Integrative Energy Engineering, Korea University, Seoul, 02841, Republic of Korea; Department of Biomicrosystem Technology and KU Photonics Center, Korea University, Seoul, 02841, Republic of Korea; Center for Opto-Electronic Materials and Devices, Post-Silicon Semiconductor Institute, Korea Institute of Science and Technology (KIST), Seoul, 02792, Republic of Korea

**Keywords:** tandem solar cells, detailed balance limit, intermediate mirror, plasmonic metamaterials

## Abstract

An intermediate mirror has been proposed to enhance multijunction solar cell efficiency by selectively reflecting the light beyond higher energy bandgap of top cell, while simultaneously transmitting the rest of lower-energy light. Therefore, it reduces the higher-energy absorption spectral tail of the bottom cell (thermalization loss) and increase the absorption in the top cell. However, its effectiveness has only been theoretically validated in simplified tandem with basic components such as an antireflection coating (ARC) and top/bottom absorbers. In contrast, experimentally optimized tandem cells, such as perovskite (PVK)/silicon (Si) two-terminal configurations, include additional stacked electrodes, ultrathinned intermediate electrode, and random textures to maximize efficiency. Herein, we revisited the role of the intermediate mirror in these advanced tandem cells. Our results show that the incorporation of ideal intermediate mirror (IIM) does not improve efficiency both in textured and flat tandem cells, with its theoretical upper limit of efficiency being similar to or even lower than that of experimentally optimized cells.

## Novelty and impact statement

An entropic loss in solar cells can be addressed using a top cell with a higher energy bandgap and a bottom cell with a lower energy bandgap (i.e., multicolor limit). A spectrally selective intermediate mirror has been proposed to enhance light management and power conversion efficiency (PCE) of such multijunction solar cells. Positioned between the top and bottom cells, this mirror selectively reflects high-energy light for the top cell, while simultaneously transmitting lower-energy light to the bottom cell, reducing thermalization loss. However, the effectiveness of intermediate mirrors has so far been validated only theoretically in simplified tandem with basic components such as an antireflection coating (ARC) and top/bottom absorbers. Recently, more advanced tandems, such as perovskite (PVK)/silicon (Si) cells, have included additional layers like stacked electrodes and random textures to maximize PCE. Herein, we revisited the effectiveness of both ideal and realistic mirrors (plasmonic nanoparticle-dispersed layers), in the latest experimentally optimized PVK/Si cells. Contrary to the previously suggested effectiveness, our results showed that even ideal intermediate mirrors (IIM) are only effective in tandem cell with relatively thick intermediate electrodes, with PCE comparable to or less than those of experimentally optimized, textured PVK/Si cells, implying new nanophotonic strategies rather than reliance of intermediate mirrors are required for tandem cells.

## 1 Introduction

To overcome the Shockley–Queisser (S-Q) limit of single-junction photovoltaic (PV) cells (∼33.5 % at 300 K) [1], there has been considerable recent interest in multijunction (tandem) PV cells [2], [3], [4]. In particular, the rapid advancements in perovskite (PVK) PV technology have led to significant progress in PVK-based tandem PV cells, such as PVK/silicon (Si) or PVK/PVK tandem cells, with experimental reports showing power conversion efficiencies (PCE) exceeding the single-junction S-Q limit, reaching up to 34.6 % [5]. However, the S-Q limit for double-junction PV cells (∼42 % at 300 K) has not yet been reached [6], and various approaches – such as optical, active material, and interfacial optimizations – are being explored to address this challenge. Among various optical optimizations, the importance of an intermediate mirror (also called a spectral splitting mirror) has been highlighted in tandem PV cells [7], [8], [9], [10], [11], [12]. A general issue in tandem PV cells is the overlap between the low-energy absorption (*A*) spectral tail of the top cell and the high-energy *A* spectral tail of the bottom cell; this is generally known as entropic or thermalization loss [9], [10], [11], [12]. Therefore, introducing an intermediate layer with spectral splitting functionality in 2-terminal (2-T) tandem PV cells, which also acts as the transparent electrode connecting the top and bottom cells, could not only reduce thermalization loss but also increase the voltage of each of the stacked cells by fully reflecting energy above the top cell’s bandgap (external luminescence, hereafter its efficiency is denoted as *η*_*ext*_) and transmitting the remaining solar spectrum to the bottom cells. Therefore, such intermediate mirror enables ideal light management in terms of thermodynamic optimization of tandem PV cells. In this context, over the past decade, quantitative analyses have demonstrated such positive optical effects of intermediate mirrors in tandem PV cells, and various nanophotonic designs for efficient intermediate mirrors have been proposed, such as stacked dielectrics (distributed Bragg reflector (DBR)), metasurfaces, and air gap [7], [8], [9], [10], [11], [12], [13].

However, over the past few years, experimental approaches that are practically accessible – such as optimizing interfacial texturing and the thickness of intermediate transparent electrodes – have led to rapid advancements in enhancing the *A* of top PVK and bottom Si cells and the resulting PCE [2], [3], [4], [14], [15], [16]. Across the solar spectrum, the intrinsic refractive index (*n*) dispersion of indium zinc oxide (IZO) or indium tin oxide (ITO), which have been widely used as intermediate transparent electrodes, is lower than that of PVK (see Figure S1, Supporting Information (SI)). As a result, these intermediate transparent electrodes are likely to reflect both higher- and lower-energy light more effectively (e.g., through total internal reflection (TIR)), which conflicts with the aforementioned ideal conditions for a spectrally selective mirror. However, controlling its thickness to be extremely thin (around 10 nm or less), but still effective for carrier transport, can minimize such impedance mismatching between PVK and intermediate layer. As such, together with advancements in introducing ARC such as magnesium fluoride (MgF_2_) on the surface of top cells, this thinning of the intermediate transparent electrode has significantly improved the *A* in both top PVK and bottom Si cells [14], [15], [16], [17]. Additionally, the implementation of the micron-scale random texturing on the both side of the bottom cell, operating in the ray-optic regime, can further minimize reflections in the low-energy regime, thus broadening the *A* spectrum of the bottom Si cells [14], [18]. This enhanced *A* of the bottom Si cell can overwhelm the possible thermalization losses, which could be caused by its broadened *A* spectrum [14], [15], [16], [17], [18].

Note that such positive effect of the spectrally selective mirroring on tandem PV cells, quantitatively suggested thus far, were based on simplified architecture of tandem cells, sequentially comprised of ARC, top/bottom absorbers, and substrate, rather than on such experimentally optimized, realistic structures [9], [11], [12]. Therefore, there is a demanding need to revisit the effectiveness of the intermediate mirror design in the latest experimentally optimized PVK/Si tandem PV cells. Moreover, previously proposed designs like DBR [7], scatterer-based metasurfaces [11], and air gaps [9] as the promising intermediate mirrors would be challenging to be readily integrated into currently available PVK/Si tandem cells, implying a need for the practically implantable intermediate mirror designs.

In this work, we numerically revalidate the effectiveness of an IIM in state-of-the-art PVK/Si tandem PV cells by combining *A* quantification (i.e., the numerical calculation hybridizing nanophotonic and ray-optic approaches) and S-Q limit analyses. We found that in tandem cells with micro-random texturing on the both side of the bottom Si cells and an ultra-thin intermediate layer (10 nm or less), introducing a spectrally selective mirror in the intermediate layer cannot lead to an increase in PCE. However, the integration of IIM can enhance both *A* and voltage, when its thickness is thick enough. Additionally, we designed a plasmonic metamaterial mirror (PMM) that could be seamlessly integrated into the existing IZO layer by a solution-dispersion of plasmonic nanoparticles (NPs), which are highly compatible with the currently accessible fabrication process for PVK/Si tandem cells.

## 2 Theoretical analysis approach for PVK/Si tandem PV cell

### 2.1 Importance of realistic modeling

**Figure 1:**
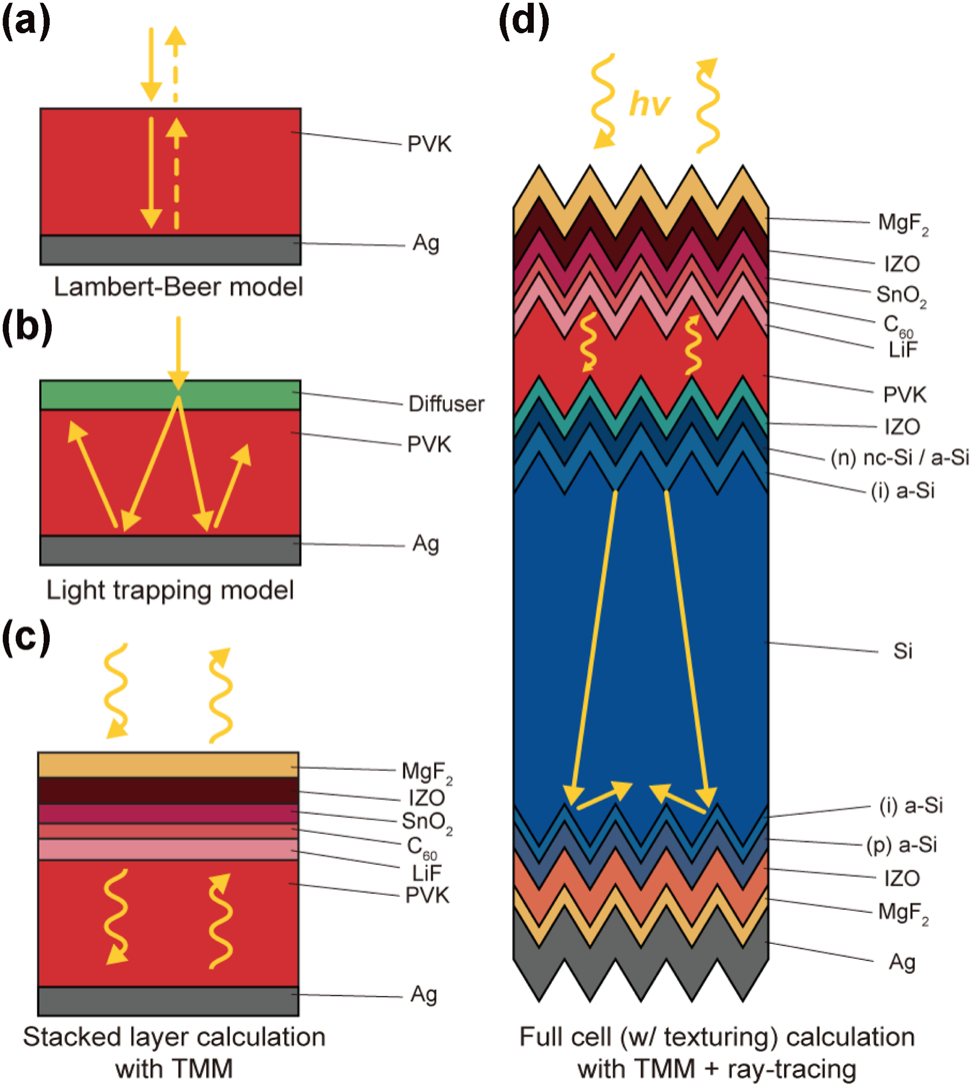
Schematic illustration of computational methods for analyzing photovoltaic (PV) cells. (a) Lambert–Beer model for simulating normal reflection of rays and their absorption in a homogeneous medium. (b) Light trapping model accounting for multiple scattering and reflection of rays. (c) Stacked layer calculations using the transfer matrix method (TMM) to analyze interference in multi-layered structures with varying thicknesses and refractive indices. (d) Full PV cell analysis incorporating ray optics and interference effects, utilizing the recently developed Rayflare model.

Theoretical studies on PV cells, conducted in this study, were collectively based on full-3D electromagnetic simulation for *A* and analytical approaches (i.e., S-Q limit analysis) for open circuit voltage (*V*_*oc*_), short circuit current (*J*_*sc*_), *η*_*ext*_, which is represented by external quantum efficiency (EQE), and PCE [19], [20], [21], [22], [23], [24], [25], [26], [27], [28], [29]. In general, the relevant previous studies, reported thus far, have been based on the assumption that solar cells are composed of a simplified single slab. Subsequently, the use of two typical models including (i) the Lambert-Beer (Figure 1a) and (ii) light-trapping (Figure 1b) models can be justified to account for *A* of solar cells according to the cell thickness, texturing, internal interference (e.g., Fabry–Perot resonant absorption), and diffusive scattering. However, both methods are unable to quantify *A* in full-cell architectures, which realistically consist of many sublayers, each with nanometer-scale thickness or even thinner (Figure 1c), as well as complex random textures operating within the ray-optic regime.

Especially, as solar cell research has advanced, both electrical (matching bandgap energy and promoting charge extractions) and optical properties (ARC coatings, internal reflection, and light interference) have been well optimized for a maximal PCE of both single- (Figure 1c) and multi-junction (Figure 1d) solar cells. In particular, PVK/Si tandem cells have been experimentally optimized by implementing various sublayers, including ARC (MgF_2_), recombination layers (IZO), selective charge transporting layers (stacked SnO_2_, C_60_, and LiF), and submicron- and micron-scale multilevel texturing on rear mirror (Ag), as shown in Figure 1d. Consequently, the simplifying such entire architecture into a single bulk layer can be no longer validated.

In this work, we developed the realistic models, reflecting the full cell structures of the latest experimentally optimized PVK/Si tandem solar cells that exhibited the benchmark PCE [15], excepting 2-(9H-carbazol-9-yl)ethyl phosphonic acid (2PACz) (Figure 1d). As 2PACz is too thin (i.e., 2 nm), its optical effect within whole cells is negligible. In general, transfer matrix model (TMM) has been the gold standard for numerical quantitation of the reflection (*R*), transmission (*T*), and *A* of the stacked dielectrics such as the cell architectures, shown in Figure 1c. However, for the randomly textured tandem cell (Figure 1d), such purely nanophotonic numerical simulation is not enough for quantifying *A* of the full cells. This is because the micron-scale textured surface needs to be analyzed within the ray-optic regime. Therefore, in this study, we carried out integrated optical modeling that combines TMM and ray-tracing (i.e., RayFlare), as described in Figure 1d [30]. The dielectric constants (Figure S2, SI) and thicknesses (Table S1, SI) of each layer in the model systems were obtained from experimental results [15]. Additional details are provided in Section 2 of SI. Our theoretical predictions of *A* and EQE (presented below) exhibit strong agreement with the corresponding experimental results [15].

### 2.2 Importance of downward external luminescence

Once we obtained *A* of each cell, we performed the analyses of the corresponding detailed balance limit of cells (S-Q limit parameters). To elucidate the role of intermediate mirror in *A* and PCE of the tandem cells, we excluded the non-radiative loss, such as Auger and Shockley-Read-Hall (SRH) recombinations. Thus, the obtained S-Q limit parameters were higher than the experimental results.

For a single-junction cell, we can calculate *J*_*sc*_, as follows [1], [9], [20], [21], [22], [23], [24], [25], [26]:

(1) $$J_{sc}=q\int a\left(E\right)S\left(E\right)dE$$

(2) $$V_{oc}=V_{oc\cdot ideal}-\frac{k_{B}T}{q}\ln\left(\frac{1}{\eta_{\mathrm{ext}}}\right)$$

(3) $$J\left(V\right)=J_{sc}-J_{\mathrm{rad}}\left(V\right)=J_{sc}-\frac{\pi }{\eta_{\mathrm{ext}}}e^{\frac{qV}{k_{B}T}}\overset{\infty }{\underset{E_{g}}{\int }}a\left(E\right)b\left(E\right)dE$$

where $S\left(E\right)$ is the solar spectrum; *q* is the electron charge; $a\left(E\right)$ is the *A* of the active layer; *k*_*B*_ is the Boltzmann constant; $b\left(E\right)$ represents blackbody radiation; *J* is the current density; *J*_*rad*_ is the radiative current density; *E*_*g*_ is the energy bandgap of the active material; and *V* is the applied voltage. The ideal open circuit voltage (*V*_*oc*⋅*ideal*_) is determined by the bandgap energy, which corresponds to the difference between *n*- and *p*-type quasi Fermi levels.

In tandem PV, the two cells form a series circuit. Thus, *J*_*sc*_ and *V*_*oc*_ of tandem cells follow the current matching and *V* distribution across the top and bottom cells, respectively. Additionally, the *η*_*ext*_ of the top cells further affects the *A* of the bottom cells. Therefore, the S-Q limit analyses should be modified as follows [6], [27], [28], [29]:

(4) $$\begin{array}{ll} J_{\mathrm{top}}\left(V_{\mathrm{top}}\right) & =J_{sc,\mathrm{top}}-J_{\mathrm{rad,top}}\left(V_{\mathrm{top}}\right)=J_{sc,\mathrm{top}}\\ & \;-\frac{\pi }{\eta_{\mathrm{ext,top}}}e^{\frac{qV_{\mathrm{top}}}{k_{B}T}}\overset{\infty }{\underset{E_{g,\mathrm{top}}}{\int }}a_{\mathrm{top}}\left(E\right)b\left(E\right)dE \end{array}$$

(5) $$\begin{array}{ll} J_{\mathrm{bot}}\left(V_{\mathrm{bot}}\right) & =J_{sc,bot}+\eta_{\mathrm{ext,top,back}}J_{\mathrm{rad,top}}\left(V_{\mathrm{top}}\right)\\ & \;-J_{\mathrm{rad,bot}}\left(V_{\mathrm{bot}}\right)\\ & =J_{sc,bot}+\eta_{\mathrm{ext,top,back}}\frac{\pi }{\eta_{\mathrm{ext,top}}}e^{\frac{qV_{\mathrm{top}}}{k_{B}T}}\\ & \;\times \overset{\infty }{\underset{E_{g,\mathrm{top}}}{\int }}a_{\mathrm{top}}\left(E\right)b\left(E\right)dE-\frac{\pi }{\eta_{\mathrm{ext,bot}}}e^{\frac{qV_{\mathrm{bot}}}{k_{B}T}}\\ & \;\times \overset{\infty }{\underset{E_{g,bot}}{\int }}a_{\mathrm{bot}}\left(E\right)b\left(E\right)dE \end{array}$$

where *a*, *V*, *J*, *J*_*sc*_, *E*_*g*_, and *η*_*ext*_ for the top and bottom cells are denoted with the subscripots “*top*” and “*bot*,” respectively. Additionally, the portion of *η*_*ext*_ from top cells that transfers to the bottom cells is indicated with the subscript “*top,back*.” The details of derivations of Equations (4) and (5) are described in Section 3 of SI.

From Equations (1)–(5), it can be seen that increasing *η*_*ext*_ in both the top and bottom cells can reduce the penalties on *V*_*oc*_ and *J*_*sc*_, thereby improving the PCE. An increase in downward luminescence *η*_*ext,top,back*_ can also enhance *J*_*bot*_ particularly at the high-energy *A* spectral tail of the bottom cell (Equation (5)). However, reducing *η*_*ext,top,back*_ can promote re-absorption and photon-recycling in the top cell, leading to improvements in both *J* and *V* of the top cell. This, in turn, raises the bar for the current-matching condition and increases the total *V*_*oc*_.

Furthermore, reducing *η*_*ext,top,back*_ decreases the *A* of high-energy photons at the spectral tail of the bottom cell, thereby minimizing thermalization loss. This enables a greater bandgap between the *n*- and *p*-type quasi Fermi levels, thus enhancing the *V*_*oc*⋅*ideal*_ for both the top and bottom cells. As a result, the overall *V* of the tandem cell increases.

**Figure 2:**
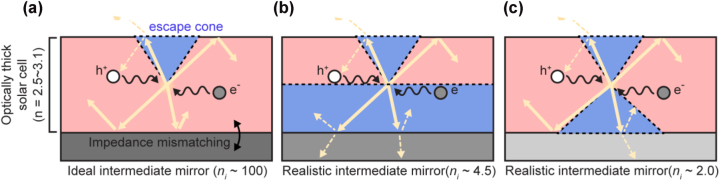
Schematics illustrating the external luminescence behavior of the top cell under varying characteristics of the intermediate layer. (a) Perfectly ideal mirrors, assuming a high-refractive-index metamaterials (HIM), can prevent luminescence light from escaping through the rear side of the top cell due to perfect reflection. (b) An intermediate layer with a high refractive index (*n*_*i*_, ∼ 4.5), higher than that of the solar cell (*n* = 2.5–3.1), creates a wide escape cone (depicted as a light blue triangle) at the rear side. (c) An intermediate layer with a low refractive index (*n*_*i*_ ∼2.0) results in a narrow escape cone at the rear side.

As shown in Figure 2, such external luminescence within the optically thick top PVK cell can be controlled by varying the type of underlying intermediate layer: (i) a perfect IMM (Figure 2a) and (ii-iii) realistically accessible intermediate layers with different refractive indices (Figure 2b and c). These three cases can be specifically designed by adjusting the refractive index of the intermediate layer (*n*_*i*_), particularly within the luminescence spectral range (or beyond the bandgap energy of the top cells).

First, in the case of an IIM, luminescence should escape into the air exclusively through the top surface (*η*_*ext,top*_), as illustrated by the escape cone in Figure 2a (bluish area). This effect of a perfect mirror can be achieved through highly mismatched impedance at the rear side of the top cell. To realize this condition, an extremely high *n*_*i*_ of 100 (corresponding to high-refractive-index metamaterials (HIM) [31], [32], [33], [34], [35], [36], [37], [38]) and an extremely low *n*_*i*_ of 0.001 (corresponding to index-near-zero (INZ) metamaterials [39]) could be utilized. Figure S3 in the SI summarizes the *R* and *T* of luminescence from the top PVK to the HIM- and INZ-based mirrors at various incident angles. Especially, inside the HIM, optical path length can be squeezed by a factor of 100 due to *n*_*i*_ of 100. As a result, the thickness of only a few tens of nanometers is sufficient to achieve near-perfect *R* when employing the HIM as the IIM. In contrast, an INZ of the same thickness does not effectively suppress luminescence escape from at the rear side of the top cell. In this ideal mirror case, the *η*_*ext*_ only through the top surface of a thick (or thin) PVK layer simplifies to $1/{n_{\mathrm{PV K}}}^{2}$ ( $1/4{n_{\mathrm{PV K}}}^{2}$ ) [9], [40].

Second, we considered realistic intermediate layers with a relatively high and low *n*_*i*_: (ii) *n*_*i*_ = 4.5 (Figure 2b) and (iii) *n*_*i*_ = 2.0 (Figure 2c), which corresponds to underneath Si and IZO, respectively. In contrast to the IIM case, these realistic intermediate layers allow luminescence to transmit to the underneath bottom cells (*η*_*ext,top,back*_), with the extent of transmission depending on their *n*_*i*_ (i.e., $1/{\left(n_{\mathrm{PV K}}/n_{i}\right)}^{2}$ for optically thick layer). Therefore, at the rear side of the top cells, a higher *n*_*i*_ expands the escape cone (approaching ∼ 90°), whereas a lower *n*_*i*_ narrows it, as shown in Figure 2b and c. This downward luminescence further affects the *J*_*sc*_, as described by Equations (5). However, in these cases of realistic mirrors, the escape cone on the top side remains unchanged compared to the IMM case. Furthermore, for *n*_*i*_ = 4.5 (Figure 2b), although the escape cone approaches to 90°, some luminescence could fail to escape through the rear side due to waveguide modes directing light toward the side walls of the cells.

Below the bandgap energy of the top cells, we used the well-known quarter-wave ARC conditions to achieve the perfect *T*. In particular, corresponding *n*_*i*_ dispersion was designed to follow $\sqrt{n_{\mathrm{PV K}}n_{Si}}$ , while its thickness was set to $\lambda /\left(4n_{i}\right)$ , where *λ* is the wavelength of interest. Given the low-absorption wavelength of Si (*λ* = 850 nm), the optimal mirror thickness was determined to be 72 nm.

## 3 Result and discussion

### 3.1 The case of textured PVK/Si tandem PV cell

**Figure 3:**
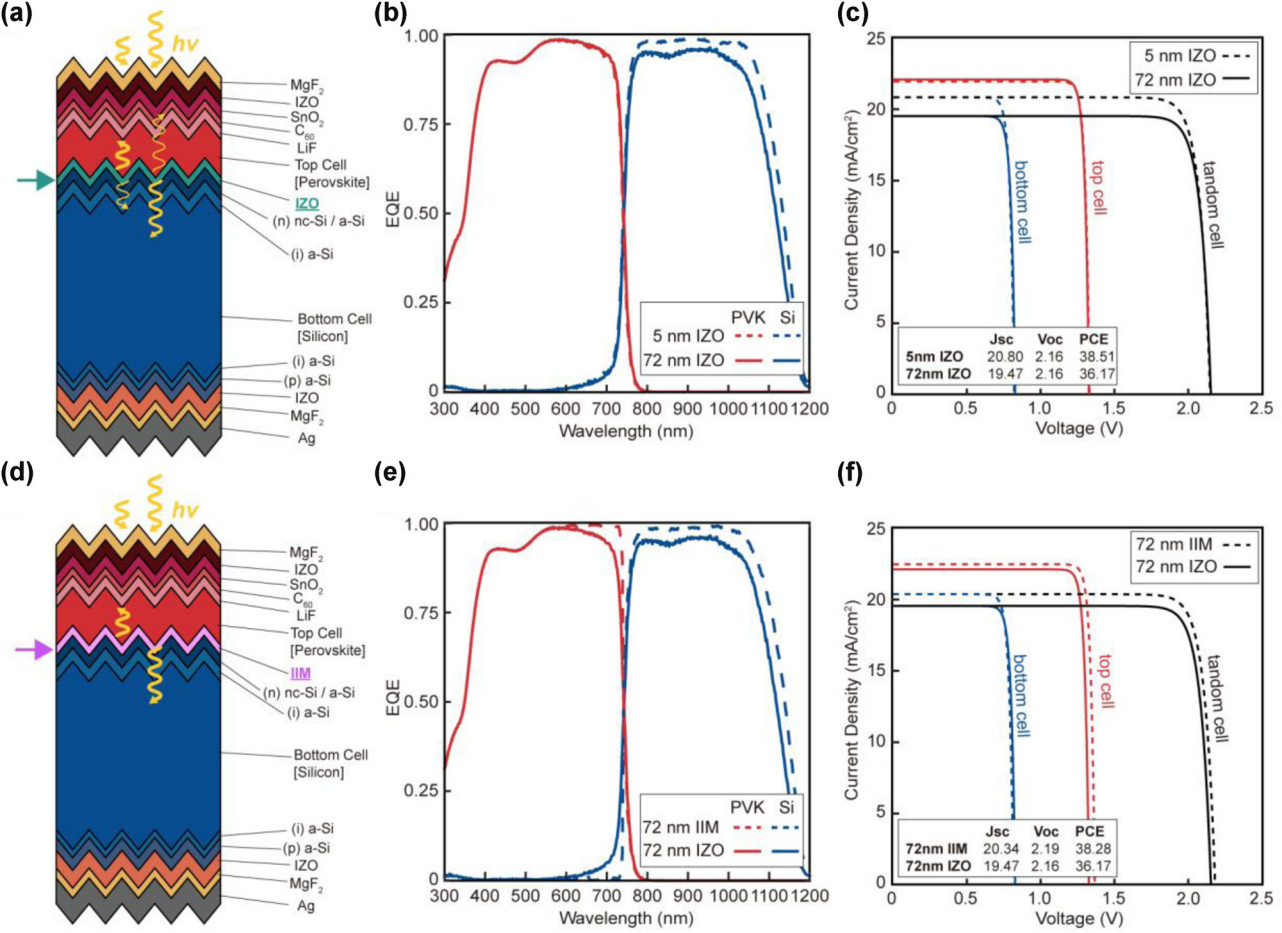
Numerical analysis for a textured perovskite (PVK)/silicon (Si) tandem PV cell. (a–c) Schematic illustration of the calculation model (a), absorption quantification (i.e., external quantum efficiency (EQE)) results (b), and Shockley–Queisser (S–Q) limit analysis (c) for a textured tandem cell with an indium zinc oxide (IZO) intermediate layer. (d–f) Schematic illustration of the calculation model (d), absorption quantification results (e), and S-Q limit analysis (f) for a textured tandem cell with an ideal intermediate mirror (IIM). Key performance parameters of PV the cell, including short circuit current (*J*_*sc*_), open circuit voltage (*V*_*oc*_), and power conversion efficiencies (PCE), are provided in the bottom left corner of (c) and (f).

We first performed numerical simulations and S-Q limit analysis on the state-of art PVK/Si tandem PV cell (Figure 3a–c). It is noteworthy that tandem PV cell with 5 nm IZO, coinciding with the experimentally optimized structure, exhibits the EQE spectra, which well matches with the experimental results [15] (Figure 3b). The corresponding detailed balance limit of cells, including *V*_*oc*_, *J*_*sc*_, and PCE, are indicated in Figure 3c.

An increase in thickness of IZO from 5 nm to 72 nm gave rise to the significant decrease in EQE (or *A*) of the bottom cell for following reason (Figure 3b). The optical effects of IZO can be negligible at a thickness of 5 nm, whereas they become significant at 72 nm in thickness. Thus, as mentioned above, the thicker IZO elucidates the TIR (see Figure S1 of SI); consequently, reducing the *T* from top to bottom cells across the solar spectrum. This effect could increase *A* and the resultant EQE of the top cells. However, as PVK can already absorb the most of incoming light, an increase in EQE was negligible (Figure 3b). By contrast, EQE of the bottom Si cells was found to decrease significantly, when the thickness of IZO increased from 5 nm to 72 nm. Even though a thicker IZO layer narrows the escape cone of downward luminescence from the top PVK layer, its impact on reducing thermalization loss remains negligible. Overall, thicker IZO showed a lower *J*_*sc*_ and the resultant PCE, while *V*_*oc*_ remains intact (Figure 3c). Indeed, the corresponding experimental report proved that thinning IZO improved PCE [15].

Next, we compared a 72 nm thick IZO with an IIM of the same thickness to revisit the effectiveness of an IIM (Figure 3d). The replacement of IZO with IIM notably improved both the *R* of a higher energy light and the *T* of a lower energy light, leading to an increase in *A* (and resultant *J*_*sc*_) of both the PVK and Si cells (Figure 3e). Furthermore, the IIM minimized *η*_*ext,top,back*_ from the PVK layer. Actually, the thermalization loss at the higher energy *A* spectral tail of Si becomes considerably reduced after introducing IIM instead of IZO. This implies that IIM can enhance *V*_*oc*_ of the total tandem cell (Figure 3f). However, it is noteworthy that a textured PVK/Si cell even with IIM (PCE of 38.28 %) cannot outperform a textured PVK/Si cell with a realistic 5 nm IZO (PCE of 38.51 %). Although an IIM can increase the *V*_*oc*_, this effect was compromised by the notable reduction of *J*_*sc*_ of the bottom Si cell.

Although our primary focus has been on reducing the entropic loss of IIM and its ability to overcome the current limitations of bottom cells using experimentally optimized cell models (e.g., with a PVK thickness of 800 nm) [15], IIM is more effective in cases where the top PVK cell does not absorb sufficient light. To explore this further, we performed calculations for cases with a thinner top PVK cell (200–600 nm) while keeping all other cell structures unchanged, as depicted in Figure S4 of the SI.

The results show that a 72 nm thick IIM can achieve a higher EQE than a 5 nm thick IZO when the top PVK is relatively thin, with this effect becoming more pronounced as the top PVK thickness decreases from 800 nm to 200 nm. Similarly, as the top PVK cell thickness is reduced within this range, the reduction in thermalization loss achieved by replacing the 5 nm thick IZO with a 72 nm thick IIM becomes more significant. However, for PVK top cells with a thickness of 400 nm or more, a 5 nm thick IZO inevitably results in a higher PCE than a 72 nm thick IIM. This is because, as discussed earlier, while IIM reduces thermalization loss and increases the *V*_*oc*_, it also decreases the broadband *A* of the bottom Si cells, thereby limiting the overall PCE.

### 3.2 The case of planar PVK/Si tandem PV cell

**Figure 4:**
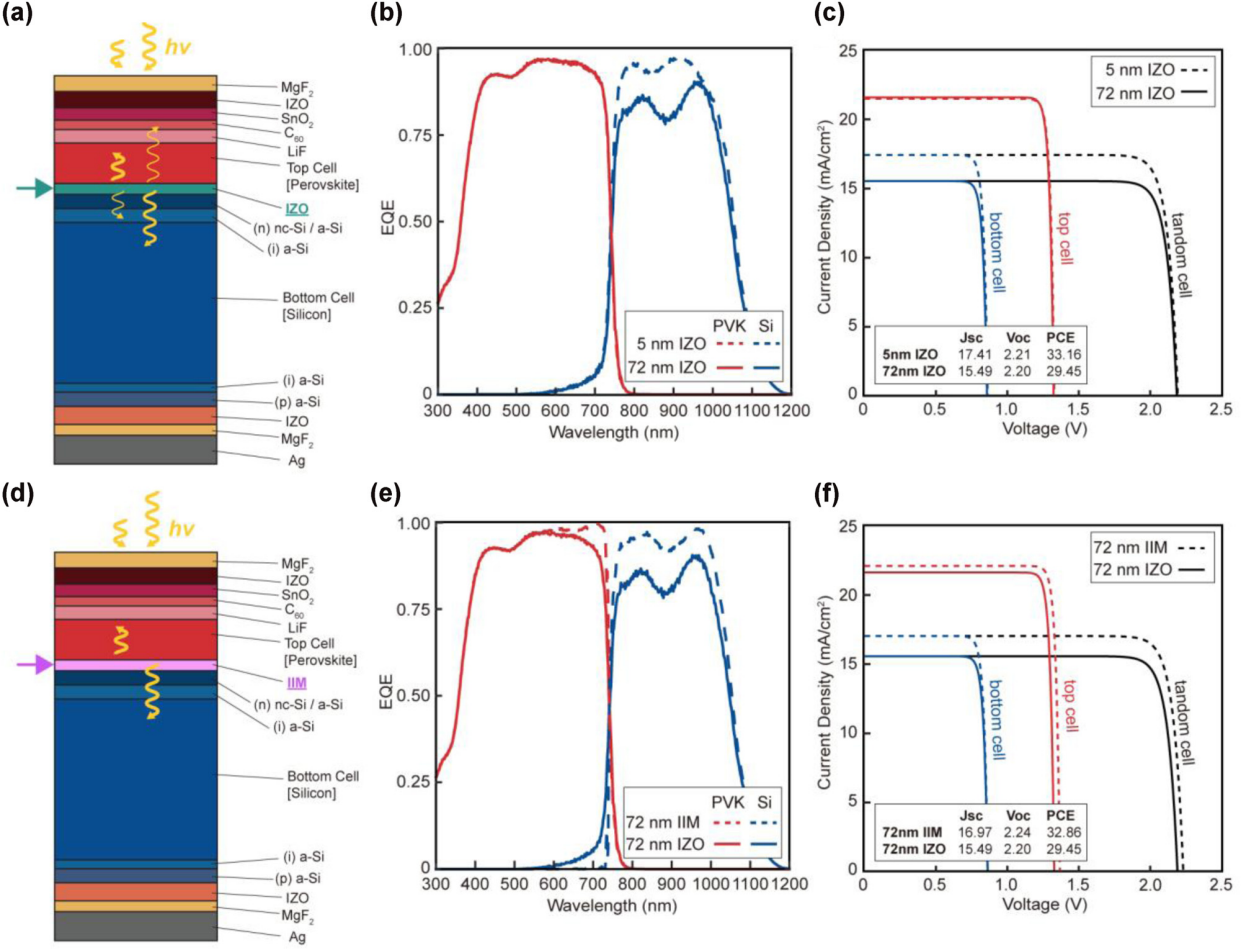
Numerical analysis for a planar PVK/Si tandem PV cell. (a–c) Schematic illustration of the calculation model (a), absorption quantification results (b), and S-Q limit analysis (c) for a planar tandem cell with an indium zinc oxide (IZO) intermediate layer. (d–f) Schematic illustration of the calculation model (d), absorption quantification results (e), and S-Q limit analysis (f) for a planar tandem cell with an IIM. Key performance parameters of PV the cell, including *J*_*sc*_, *V*_*oc*_, and PCE, are provided in the bottom left corner of (c) and (f).

Then, we evaluated whether IIM can enhance the PCE of a planar tandem PV cell (Figure 4a), which offers a more easy-to-craft platform compared to its randomly textured counterpart. As with the textured case, we first examined the effect of IZO layer thickness on the S-Q parameters (Figures 4b and c). As expected, increasing the thickness of the IZO layer from 5 nm to 72 nm selectively reduces the EQE of the Si cells, while the EQE of the PVK cells remains nearly unchanged (Figure 4b). Therefore, this leads to a decrease in both *J*_*sc*_ and PCE of the entire cells (Figure 4c).

As shown in Figure 4d–f, using an IIM of the same thickness (72 nm) improves the EQE (or *A*) of both cells while minimizing thermalization loss (i.e., reducing *η*_*ext,top,back*_ and increasing *V*_*oc*_). As a result, we observed an enhancement in both *J*_*sc*_ and *V*_*oc*_ of the entire cells, as presented in Figure 4f. Overall, the PCE of the planar PVK/Si tandem cell can increase from 29.45 to 32.86 % when replacing IZO with IIM. However, this achievable PCE of the 72 nm thick IIM-integrated flat PVK/Si tandem cells (32.86 %) remains slightly lower than that of its 5 nm thick IZO-integrated counterpart (33.16 %).

### 3.3 Use of plasmonic metamaterial metamirror (PMM)

One practical approach for integrating a spectrally selective mirror into PVK/Si tandem cells is the simple dispersion of plasmonic NPs within the IZO layer to unnaturally modify its effective *n* (*n*_*eff*_)–a concept we refer to as the plasmonic metamaterial mirror (PMM). Plasmonic NPs, typically tens of nanometers in size, exhibit strong scattering and near-field enhancement in the visible range (beyond the higher energy bandgap) owing to localized surface plasmon resonance (LSPR). This enhanced dipolar resonances via LSPR can increase the electric polarization and the resulting *n*_*eff*_ of the host medium in both the resonant visible and off-resonant near-infrared (NIR) regimes [32], [33], [34], [35]. Recently, we experimentally validated this theoretically suggested concept of “high-index plasmonic metamaterials” by colloidal NP self-assembly [36], [37], [38].

Regarding the design of the PMM, we selected silver (Ag) rather than gold due to its plasma frequency being below 400 nm (see also Figure S5 of the SI). The use of Ag minimizes the imaginary part of *n*_*eff*_ especially at wavelengths longer than the bandgap of the top PVK cells. Herein, we obtained *n*_*eff*_ of the corresponding IZO-based effective medium using the *s*-parameter retrieval method [32], [33], [34], [35]. The thickness of PMM was fixed at 72 nm, consistent with the previous cases.

**Figure 5:**
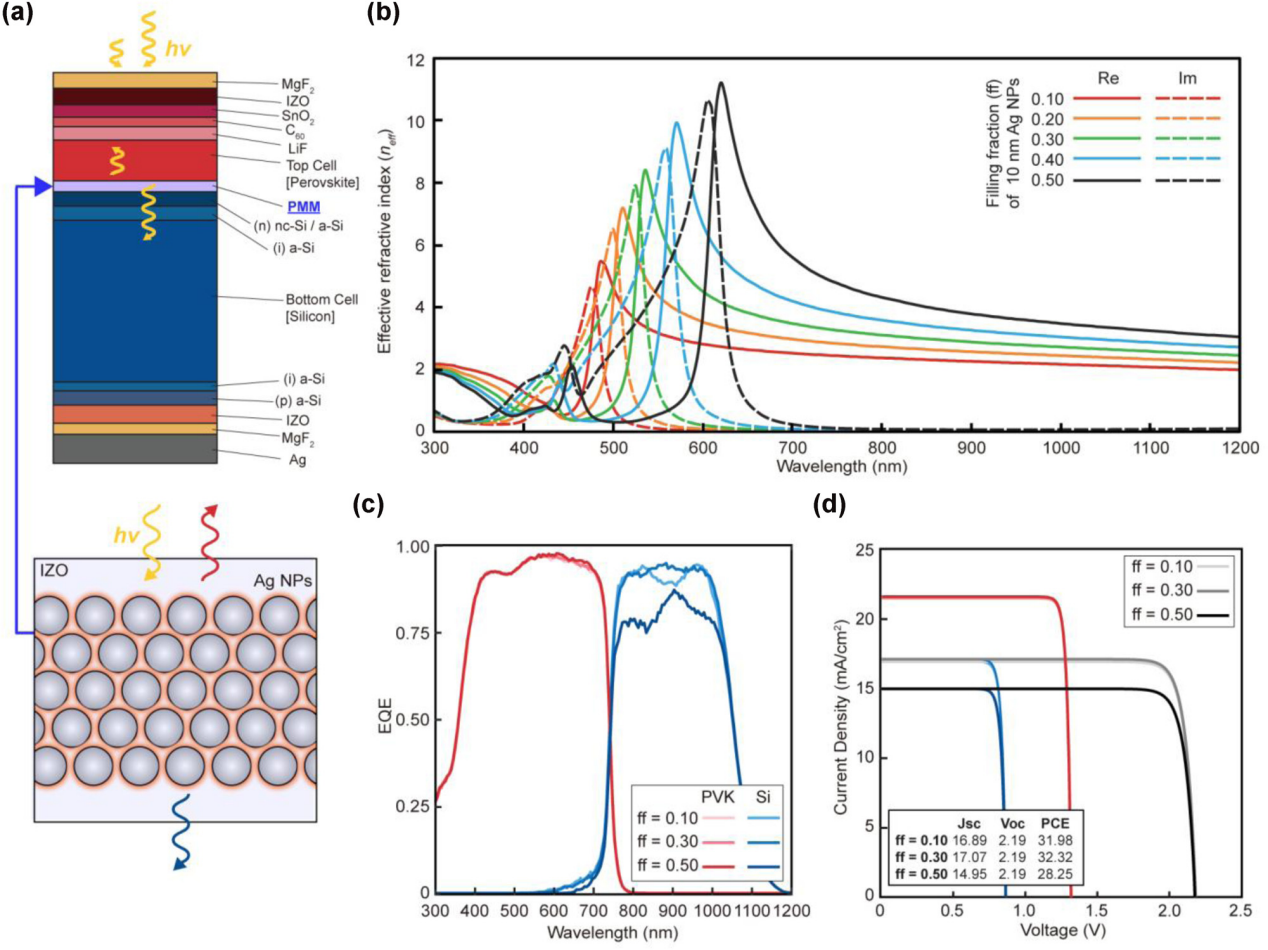
Numerical analysis for a planar PVK/Si tandem PV cell with a plasmonic metamirror (PMM) as the intermediate layer. (a) Schematic representation of the calculation model and PMM, designed by dispersing silver nanoparticles (Ag NPs) with IZO layer. (b) Effective refractive index (*n*_*eff*_) of the PMM under varying filling fraction of Ag NPs. Real (Re) and imaginary (Im) part of *n*_*eff*_ were depicted as solid and dashed lines, respectively. (c–d) Absorption quantification results (c) and S-Q limit analysis (d) for a planar tandem cell with the PMM as the intermediate layer. Key performance parameters of PV the cell, including *J*_*sc*_, *V*_*oc*_, and PCE, are provided in the bottom left corner of (d).

To optimize the PMM, we first identified the ideal NP size under a fixed filling fraction to achieve the highest *n*_*eff*_ of PMM at the higher energy bandgap of the top PVK cells – where a high *n*_*eff*_ contrast between the top PVK cell and PMM is required. Based on the calculated *n*_*eff*_ in Figure S6, we chose 10 nm Ag NPs as the optimal size. We then investigated the effect of the Ag NP filling fraction on *n*_*eff*_ (Figure 5b). The dispersion of Ag NPs (Figure 5a) can unnaturally increase the *n*_*eff*_ of IZO at the resonant wavelength. These resonant peaks of unnaturally high *n*_*eff*_ and its spectral position can be precisely tuned around at the higher energy bandgap by adjusting the filling fraction of Ag NPs (0.1–0.5). In the off-resonant regime (i.e., below the wavelength of the lower bandgap energy), the *n*_*eff*_ of PMM ranges between the *n* of the PVK and Si layers. Therefore, the PMM acts as a spectrally selective mirror, enhancing spectrally selective *R* at higher energies while improving *T* at lower energies.

It is important to note that the filling fraction of Ag NPs was limited to 0.5 to minimize undesirable A enhancement of PMM in the lower energy regime. Despite this limitation, the non-negligible imaginary part of *n*_*eff*_at the lower bandgap energy leads to a reduction in the *A* of the bottom Si layer, ultimately compromising *J*_*sc*_. This effect becomes more elucidated at higher Ag NP filling fractions (Figure 5b), as the resonant wavelengths of both the real and imaginary parts of *n*_*eff*_ undergo a redshift according to the Kramers–Kronig relation. Thereby, a PMM with a high Ag NP-filling fraction reduces *A* and the EQE of the bottom Si cells, especially in the wavelength range at where thermalization losses occur (Figure 5c and d).

Also, it is noteworthy that regardless of the Ag NP filling fraction, PMM tends to reduce *V*_*oc*_ to 2.19 V, which is lower than the values achieved by 72 nm thick IIM (2.24 V) and IZO-integrated cells (2.20 V), because the *n*_*eff*_ of PMM remains relatively high (i.e., *n*_*eff*_ ∼ 3) compared to that of the PVK layer at a wavelength of 740 nm. As a result, as already discussed in Section 2.2, PMM enhances *η*_*ext,top,back*_, leading to a reduction in *V*_*oc*_ of the entire cell. However, PMM is better than an IZO or IIM of the same thickness, in terms of promoting downward *T* from the top to bottom cells. This is because its *n* is more close to that of PVK, reducing TIR. As a result, 72 nm thick PMM-based tandem cells showed a higher *J*_*sc*_ than IZO- and IMM-based counterparts. Overall, the use of PMM as the intermediate layer results in an improved PCE of 32.32 %, outperforming the 29.45 % PCE achieved with an IZO layer and approaching the 32.86 % obtained with IMM layer of the same thickness. These results confirm the effectiveness of the designed PMM in enhancing PCE of realistic planar PVK/Si tandem PV cells.

## 4 Conclusion and outlook

Taken together, we revisited the role of the intermediate mirror in the latest advanced PVK/Si tandem cells and proposed the following key insights. Even perfect IIM is effective only in relatively thick intermediate electrodes, achieving PCE comparable to or lower than those of experimentally optimized (textured) PVK/Si tandem cells. This result is in stark contrast to previous suggestions regarding the effectiveness of intermediate mirrors. Moreover, previously proposed nanophotonic candidates for ideal intermediate mirrors, such as airgap [9], dielectric directional scatterers (metasurfaces) [11], and DBRs [7], would be challenging for seamless and practical integration into PVK/Si tandem cells. To overcome these technological challenges, we proposed a plasmonic NP-dispersed intermediate electrode as a spectrally selective mirror. Simply dispersing plasmonic NPs into the host medium enables a resonant increase in the *n*_*eff*_, which is highly compatible with the current fabrication processes of PVK/Si tandem PV cells. More critically, by adjusting the size and elements of plasmonic NPs, we can precisely control the spectral peak position of this unnaturally enhanced *n*_*eff*_ to align with the higher or lower bandgap energy, optimizing *n*_*eff*_mismatch at each bandgap. This plasmonic NP metamaterial approach enables selective enhancement of *n*_*eff*_ at the bandgap energies of the top and bottom cells, making their mirror functionality more favorable for achieving an IIM. Indeed, we demonstrated that the PCE of a flat PVK/Si tandem cell can be improved merely by randomly dispersing plasmonic NPs within currently optimized intermediate transparent electrodes. These theoretical blueprints can promote the realization of tandem cells that more closely approach the S-Q limit.

## Supplementary Material

Supplementary Material Details
