# Supplementary material for: Are nanophotonic intermediate mirrors really effective in enhancing the efficiency of perovskite tandem solar cells?
Source: Nanophotonics. 2025 Apr 9;14(8):1239–48. doi: 10.1515/nanoph-2024-0658 (PMC12019946; doi:10.1515/nanoph-2024-0658)
Supplement: Supplementary file 1 — Supplementary Material Details [file j_nanoph-2024-0658_suppl_001.pdf]

Supporting Information for

Are nanophotonic intermediate mirrors really effective in enhancing the efficiency of perovskite tandem solar cells?

Kwangjin Kim[1,+], ***Jieun Lee***[1,+], ***Jaewon Lee***[1,+], ***Jin-Young Kim***[2], ***Hae-Seok Lee***[3,4], and ***Seungwoo Lee***[1,4,5,6*]

[1]KU-KIST Graduate School of Converging Science and Technology, Korea University, Seoul 02841, Republic of Korea

[2]Department of Materials Science and Engineering, Seoul National University, Seoul 08826, Republic of Korea

[3]Graduate School of Energy and Environments (Green School), Korea University, Seoul 02841, Republic of Korea

[4]Department of Integrative Energy Engineering, Korea University, Seoul 02841, Republic of Korea

[5]Department of Biomicrosystem Technology and KU Photonics Center, Korea University, Seoul 02841, Republic of Korea

[6]Center for Opto-Electronic Materials and Devices, Post-Silicon Semiconductor Institute, Korea Institute of Science and Technology (KIST), Seoul 02792, Republic of Korea

[*]Email: seungwoo@korea.ac.kr

[+]equally contributed to this work

Keywords: Tandem solar cells, Detailed balance limit, Intermediate mirror, Plasmonic metamaterials

Contents:

1. **Dielectric constants used for numerical simulations**
2. **Numerical simulation details**
3. **Derivation of S-Q limit equations for tandem cell**
4. **Design of ideal intermediate mirror (IIM)**
5. **Effect of IIM on efficiency of cells with varying thickness**
6. **Engineering of refractive index by using plasmonic metamaterial**

1. Dielectric constants used for numerical simulations

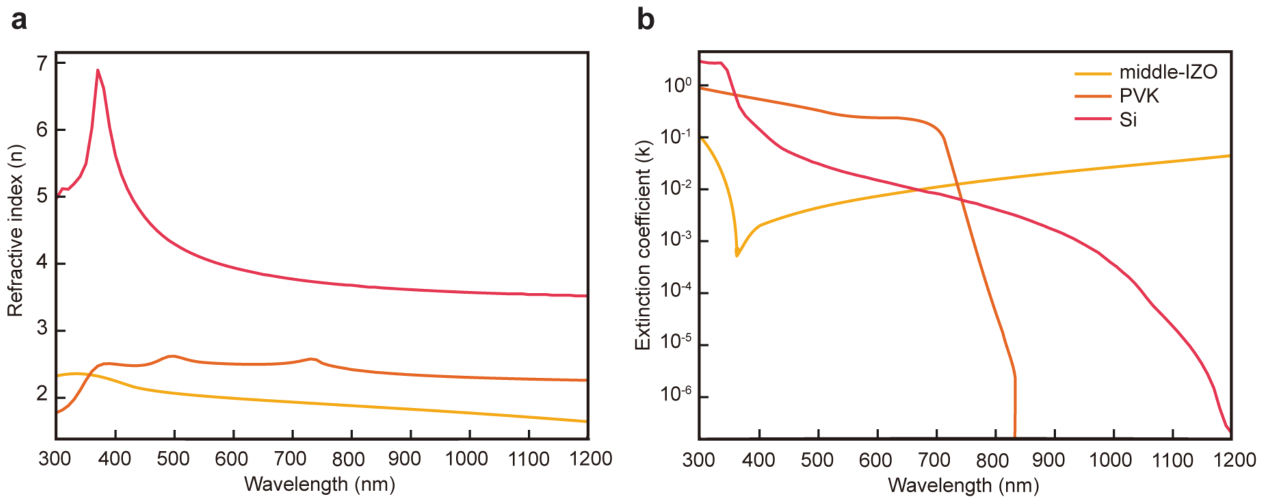

Figure S1. Refractive index (n) and extinction coefficient (k) spectra of IZO, PVK, and Si, which were used in the numerical simulations. (a-b) n (a) and k (b) of middle-IZO, PVK (Perovskite), and silicon (Si) layers across the solar spectrum. The relatively large n mismatch between IZO and Si across the spectrum enhances reflection of lower energy light at their boundary. On contrary, the relatively small n mismatch between IZO and PVK promotes transmission of higher energy light. This behavior conflicts with the effective light management and optimization of optical design in the tandem photovoltaic (PV) cells.

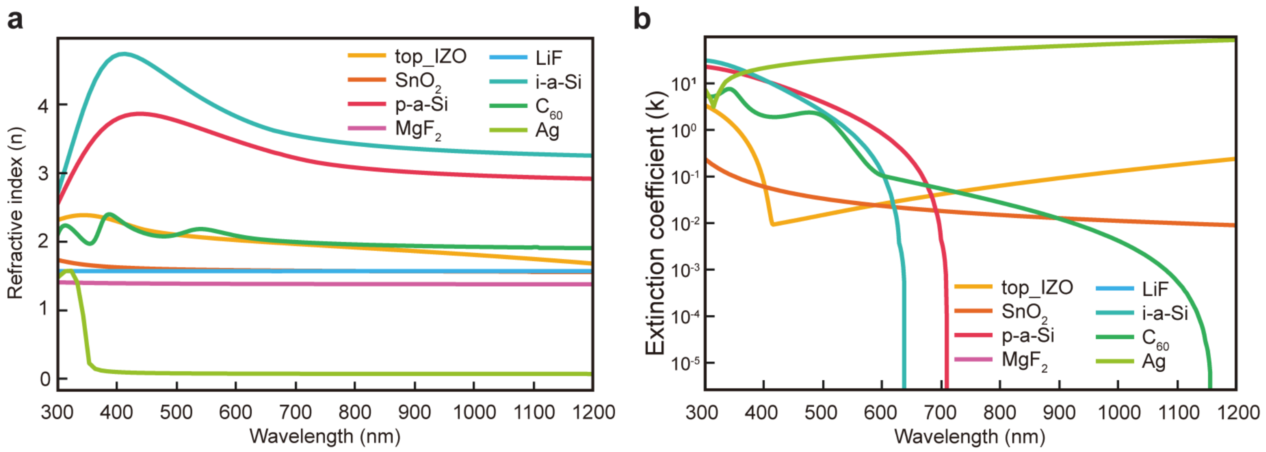

Figure S2. n and k spectra of materials used in the tandem solar cell simulations. (a-b) n (a) and k (b) of top-IZO, SnO_2, p-a-Si, MgF_2, LiF, i-a-Si, C_{60}, and Ag layers.

2. Numerical simulation details

The simulations covered a wavelength range from 300 to 1200 nm, with 900 data points evenly spaced within this range. Ray-tracing parameters included a ray count determined by $4 \cdot nx^2$, where nx was set to 30, a depth spacing of 1 nm, and randomized surface conditions enabled to simulate realistic imperfections. The energy conservation threshold was set to $I_{thresh} = 10^{-3}$, sufficient for numerical stability and convergence.

The materials used in the simulations were defined using wavelength-dependent refractive index (n) and extinction coefficient (k) data obtained from experimental sources. These data were interpolated to match with the simulation wavelength range for improved accuracy. The intermediate layer, positioned between the PVK and Si layers, was varied among three materials: IZO, PMM, and IIM. Each intermediate layer had a fixed thickness of 5 nm or 72 nm. In the case of IIM, the cut-off wavelength for the layer was set to 740 nm, corresponding to the bandgap of the PVK. For wavelengths shorter than this cut-off, the layer reflected all incident light to enhance absorption (A) in the PVK top cell. For longer wavelengths, an anti-reflection coating (ARC) was implemented to optimize transmission (T) into the bottom Si cell and minimize reflective losses.

Ray-tracing simulations were conducted under both coherent and incoherent conditions. Coherent calculations utilized the transfer-matrix method (TMM) for thin layers, which can reflect interference effects, were accurately modeled, while incoherent calculations used ray-tracing for thicker layers to capture geometric optics and scattering effects. Rayflare, the simulation tool used in this study, can integrate these two methods dynamically. TMM was applied to thin layers where interference dominates, while ray-tracing was used for thick layers such as Si, where scattering and geometric effects are more significant.

For each wavelength, reflection (R), T, and A were calculated, with A data further resolved into contributions from individual layers $(A_{per\ layer})$ and interfaces $(A_{per\ interface})$. Energy conservation $(R + T + A) = 1)$ was verified across all simulations to ensure physical consistency.

These calculations facilitated a detailed understanding of the optical performance of each intermediate layer configuration, including its impact on A, T, and R in both the top PVK cell and bottom Si cell.

Table S1. Thicknesses of each layer in the PVK/Si tandem PV cell used for the numerical simulations and analyses.

MgF_2	100 nm
Top IZO	40 nm
SnO_2	10 nm
C_{60}	12 nm
LiF	1 nm
Perovskite	800 nm
Intermediate Layer (varied)	72 nm
nc_n_Si	20 nm
i_a_Si	8 nm
Si	250,000 nm
i_a_Si	8 nm
p_a_Si	8 nm
Middle IZO	5 nm
MgF_2	200 nm

3. Derivation of S-Q limit equations for tandem cell

According to the S-Q limit, relations between J and V can be described by simple equations.

$$J(V) = J_{sc} - J_{rad}(V) - J_{nrad}(V) = J_{sc} - \frac{1}{\eta_{ext}} J_{rad}(V)$$

As we excluded the non-radiative recombination channels in solar cell, η_{ext} is determined by radiative recombination rate escaping from front side ($J_{rad,front}$) and back side ($J_{rad,back}$). Then, we can achieve the following equations:

$$\eta_{ext} = \frac{J_{rad,front}}{J_{rad,front} + J_{rad,back}}$$

$$J_{rad} = J_{rad,front} + J_{rad,back}$$

$$J_{rad,back} = \left(\frac{1}{\eta_{ext}} - 1\right) J_{rad} = \eta_{ext,back} J_{rad}$$

Finally, we find S-Q limit equations for tandem cells.

$$J_{top}(V_{top}) = J_{sc,top} - J_{rad,top}(V_{top})$$

$$\begin{aligned} J_{bot}(V_{bot}) &= J_{sc,bot} + J_{rad,top,back}(V_{top}) - J_{rad,bot}(V_{bot}) \\ &= J_{sc,bot} + \eta_{ext,top,back}\, J_{rad,top}(V_{top}) - J_{rad,bot}(V_{bot}) \end{aligned}$$

$J_{rad}(V)$ is related to external luminescence as follows:

$$J_{rad}(V) = J_{sc} - \frac{\pi}{\eta_{ext}} e^{\frac{qV}{k_B T}} \int a(E) b(E) dE$$

4. Design of ideal intermediate mirror (IIM)

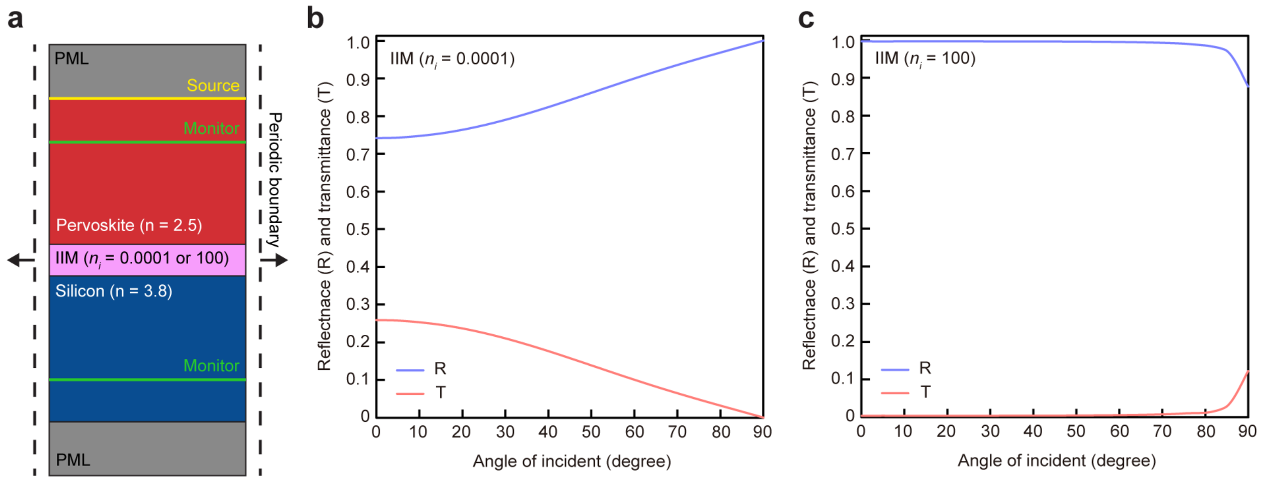

Figure S3. Full-wave numerical analysis of an ideal intermediate mirror (IIM) based on index-near-zero (INZ) and high-refractive-index metamaterials (HIM). (a) Schematic illustration of the full-wave numerical calculation model. Numerical analysis was performed at 740 nm excitation, corresponding to the bandgap of the top PVK cell. (b–c) Reflectance and transmittance spectra for a plane wave propagating from the top PVK layer to the bottom Si layer with an INZ intermediate mirror (b) and a HIM intermediate mirror (c), each with a thickness of 72 nm. The HIM layer effectively suppresses transmission from the PVK to the Si layer over a wide range of incident angles, significantly reducing luminescence escape through the rear side of the top cell. On the contrary, the INZ layer does not suppress light transmission effectively.

5. Effect of IIM on efficiency of cells with varying thickness

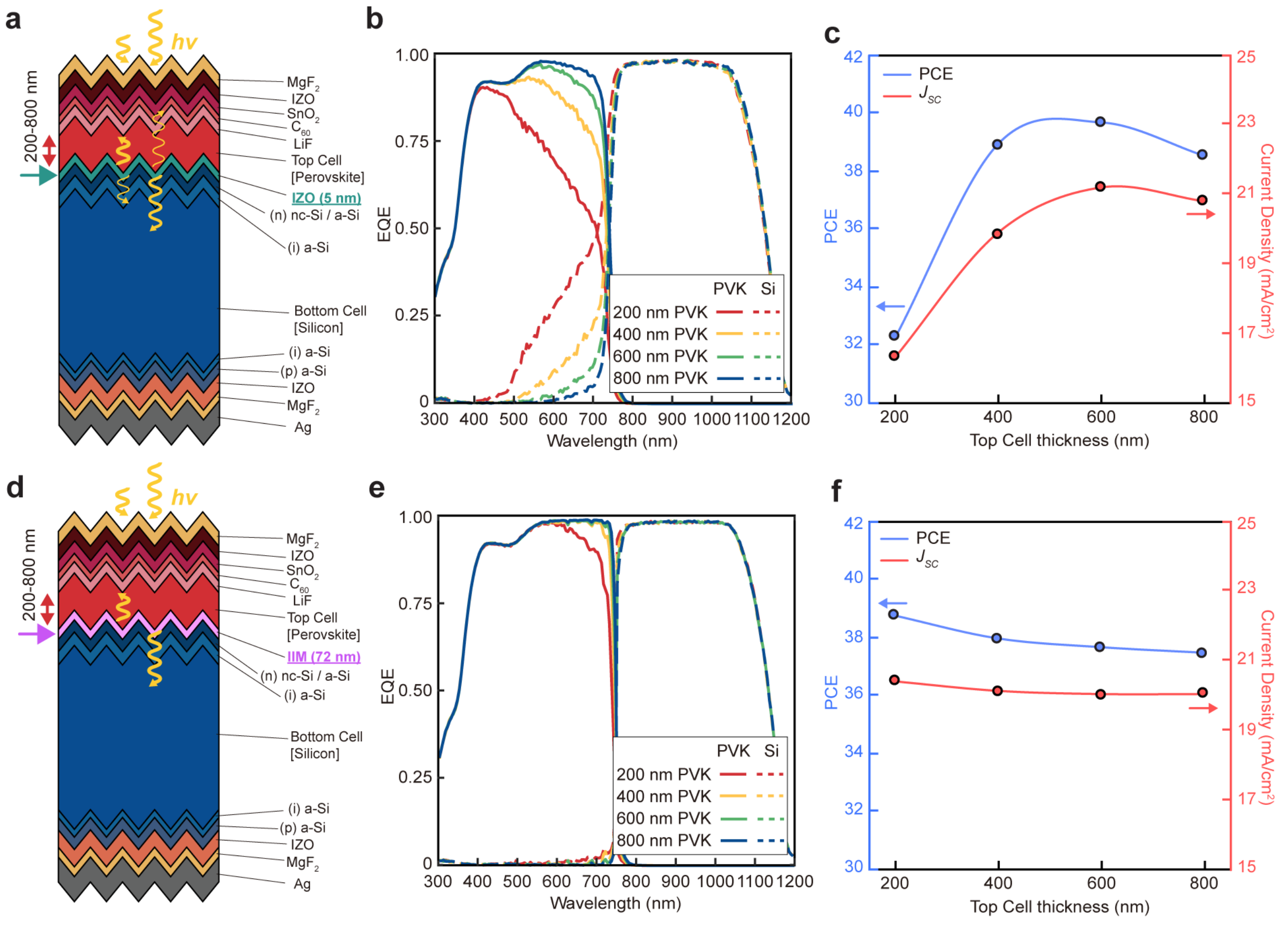

Figure S4. Numerical analysis for a textured PVK/Si tandem PV cell under varying thickness of PVK layer (top cell). (a-c) Schematic illustration of the calculation model (a), absorption quantification results (b), and S-Q limit analysis (c) for a textured tandem cell with an IZO intermediate layer. (d-f) Schematic illustration of the calculation model (f), absorption quantification results (e), and S-Q limit analysis (f) for a textured tandem cell with an IIM.

Figure S4 demonstrates the effect of the top PVK cell thickness in EQE, PCE, and J_{sc} of a textured tandem cell. With a 5 nm IZO layer, maximum PCE was achieved, when the top PVK cell thickness was reduced to 600 nm. This improvement resulted from the creation of a spectral tail in the bottom cell, which absorbed short wavelengths, leading to more balanced current generation between the two cells. As a result, the largest total J_{sc} was obtained with a 600 nm top PVK cell thickness, improving the overall PCE. This result suggests that the top PVK cell thickness should be carefully optimized not only to maximize A but also to enhance overall PCE by accounting for the bottom cell's J generation. As the top PVK cell thickness decreases, the PCE drop can be clearly observed due to the reduced J in the top cell.

When the IIM was used as the intermediate layer, efficient spectral splitting was achieved, further enhanced by the textured structure that increased the available optical path length. This resulted in high PCE even at the thinnest top PVK cell thickness. Overall, although the PCE was slightly lower compared to the tandem cell with a 5 nm IZO intermediate layer, the IIM structure prevented a significant PCE drop by better matching the current between the two cells.

6. Engineering of refractive index by using plasmonic metamaterial

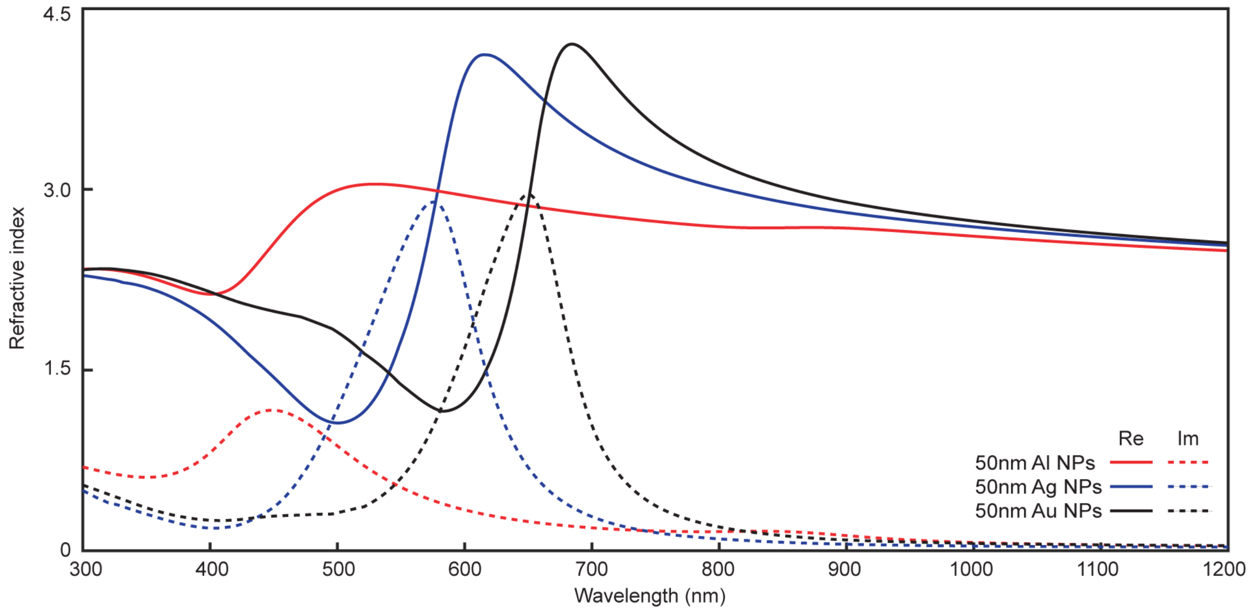

Figure S5. Real (*Re*) and imaginary (*Im*) components of the effective refractive index (n_{eff}) for plasmonic metamirror (PMM) containing 50 nm aluminum (Al), silver (Ag), or gold (Au) nanoparticles (NPs) dispersion in IZO medium. Ag NPs were chosen for designing PMM used as the intermediate layer in PVK/Si tandem cell because they allow modulation of *n* over a wider range compared to Al NPs, while keeping the resonances and resulting absorption peaks further from the long-wavelength region compared to Au NPs.

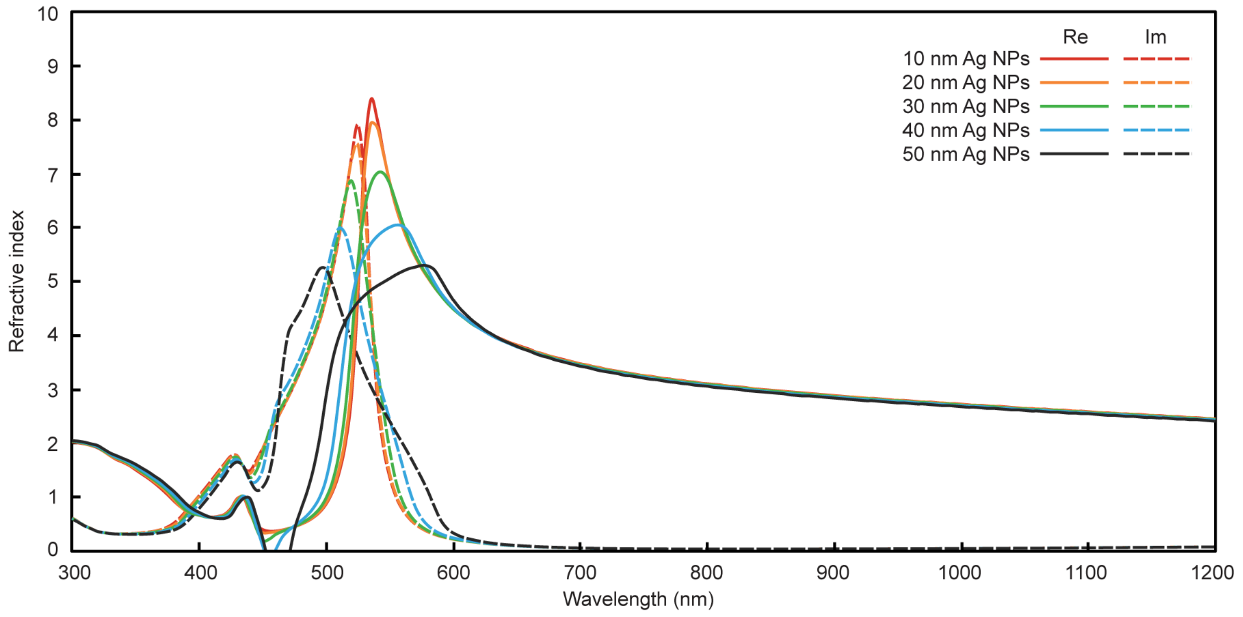

Figure S6. *Re* and *Im* components of the n_{eff} for plasmonic metamirror (PMM) composed of Ag NPs dispersions in IZO medium, with NP sizes varied while keeping the filling fraction fixed at 0.30. The 10 nm Ag NPs were chosen for designing the PMM as the intermediate layer in PVK/Si tandem

cell because they provide the highest n_{eff} in the short-wavelength region, where a high impedance mismatch between the intermediate layer and PVK is required.
